# Phages infecting the pathobiont *Escherichia coli* LF82

**DOI:** 10.1128/mra.00581-25

**Published:** 2025-08-25

**Authors:** Joanna P. Steczynska, Mia V. Ringholz, Michael J. Wannemuehler, Nick T. Peters, Kelly P. Williams

**Affiliations:** 1 Systems Biology Department, Sandia National Laboratories 111651, Livermore, California, USA; 2 Undergraduate Microbiology program, Iowa State University 1177, Ames, Iowa, USA; 3 Department of Veterinary Microbiology and Preventive Medicine and the Nanovaccine Institute, Iowa State University 1177, Ames, Iowa, USA; 4 Department of Plant Pathology, Entomology and Microbiology, Iowa State University 1177, Ames, Iowa, USA; Queens College Department of Biology, Queens, New York, USA

**Keywords:** bacteriophage, *Escherichia coli*, pathobiont, AIEC

## Abstract

*Escherichia coli* LF82 is a gut pathobiont commonly found in Crohn’s disease patients and a candidate for phage therapy. We report isolation of lytic phages able to infect LF82, with 19 unique genome sequences falling into four species clusters.

## ANNOUNCEMENT

Phage therapy is a viable treatment for inflammatory bowel disease, directly attacking any bacterial pathobiont that may characterize the incidence (1–3). One such pathobiont is LF82, an adherent invasive *Escherichia coli* (AIEC) strain isolated from a Crohn’s disease patient (4, 5). Key to successful phage therapy against LF82 relatives will be the availability of diverse phages from which to design a cocktail (2). Here, we present genome sequences for 19 phages that infect LF82, falling into four species.

Soil samples (over 100, collected and pooled throughout 2023) from Iowa State University Compost facility in Ames, IA, were suspended in phage buffer (6). Each supernatant was filtered using a 0.22-µm filter and assayed for lytic activity using the standard double-agar overlay with all growth in LB medium at 37°C (Fisher Scientific). Overnight cultures of LF82 (CU651637, obtained from Kenneth Simpson, Cornell University) were incubated with filtrate for 20 min at room temperature, then mixed with molten top agar. Plaques of varied size and clarity were obtained the next day; 36 were plaque-purified three times. Genomic DNA was extracted (Phage DNA Isolation kit, Norgen Biotek) from high titer stocks (>10^7^ pfu/mL). Libraries (DNA Prep kit, Illumina) were sequenced using the MiSeq V3 150-cycle kit (Illumina) in single-read mode.

Reads were obtained for 34 of the samples and assembled using SPAdes (7) v3.15.5, circularizing the longest contig by trimming one of the ~55 bp terminal repeats typically left for DNA circles by SPAdes. Two rejected exceptions were the LF82-26 and LF82-38 samples, whose only phage sequence was a circular form of a spontaneously induced 38.7-kbp LF82 prophage, identified by TIGER (8) as 000284495.39.torT|torS (accession CU651637, coordinates 998948–1037629). This prophage accompanied the newly isolated phage in 14 of the remaining assemblies. Some of the resulting genome sequences were identical, leaving 19 unique sequences that fell into three groups of size ~170, 53, or 42 kbp (Table 1). To determine taxonomy, a BLASTN (9) v2.16.0 + database was prepared from the 8630 Genbank accessions of taxonomically treated prokaryotic viruses listed in the International Committee on Taxonomy of Viruses (ICTV) document MSL40.v1 (10). Each 170-kbp and 42-kbp phage produced at least one BLASTN hit to these reference genomes with bitscore >10,000, and each 53-kbp phage produced a hit with bitscore >3,000 (Table 1). The 24 reference accessions associated with these hits were submitted along with our 19 new genomes to the VIRIDIC website (11) which placed the 170-kbp phages into two novel (lacking ICTV reference genomes) species clusters of the genus *Mosigvirus*, and the 42-kbp phage into a novel species cluster of the genus *Bonnellvirus*. The 53-kbp phages (best-matching ICTV reference genome from the family *Drexlerviridae*) fell into a novel species cluster and a novel genus cluster. Pharokka (12) (v1.7.5, flags: -g prodigal --dnapler) was used for gene annotation. Lack of integrase and repressor genes indicative of temperate phages suggests that all phages are virulent. Default parameters were used for all software unless otherwise specified.

**TABLE 1:** Characteristics of new unique phage genome sequences, sorted by size. ND (not determined): this genome cluster contained no ICTV reference genomes.

Phage	No. of identical genomes	Genus cluster	Species cluster	Top ICTV BLASTN hit	Reads	Size (bp)	Coverage	GC content (%)	Protein genes	tRNA genes	Genbank accession	BioSample accession
LF82-04	1	*Mosigvirus*	1	MK962757	469,574	171,304	165.6	37.56	273	1	PV660448	SAMN48508507
LF82-09	0	*Mosigvirus*	1	MK962757	384,213	170,850	154.0	37.61	271	10	PV660452	SAMN48508511
LF82-01	4	*Mosigvirus*	1	MK962757	457,885	169,999	177.6	37.62	271	2	PV648359	SAMN48508504
LF82-02	0	*Mosigvirus*	1	MK962757	360,580	169,999	145.3	37.62	271	2	PV648360	SAMN48508505
LF82-03	0	*Mosigvirus*	1	MK962757	379,706	169,999	155.8	37.62	271	2	PV660447	SAMN48508506
LF82-05	0	*Mosigvirus*	1	MK962757	395,606	169,999	159.9	37.62	271	2	PV660449	SAMN48508508
LF82-07	0	*Mosigvirus*	1	MK962757	309,138	169,999	124.2	37.62	271	2	PV660450	SAMN48508509
LF82-08	0	*Mosigvirus*	1	MK962757	466,078	169,999	187.2	37.62	271	2	PV660451	SAMN48508510
LF82-16	0	*Mosigvirus*	1	MK962757	343,464	169,999	136.3	37.62	271	2	PV660456	SAMN48508515
LF82-31	0	*Mosigvirus*	1	MK962757	413,366	169,999	168.5	37.62	271	2	PV660459	SAMN48508518
LF82-33	0	*Mosigvirus*	1	MK962757	409,507	169,999	166.0	37.62	271	2	PV660460	SAMN48508519
LF82-35	0	*Mosigvirus*	1	MK962757	460,236	169,999	184.6	37.62	271	2	PV660461	SAMN48508520
LF82-41	0	*Mosigvirus*	2	KT184309	383,771	169,675	157.3	37.68	273	2	PV660463	SAMN48508522
LF82-11	1	ND	3	KX130668	433,174	52,979	31.7	49.64	91	1	PV660454	SAMN48508513
LF82-12	4	ND	3	KX130668	344,667	52,979	47.2	49.64	91	1	PV660455	SAMN48508514
LF82-40	0	ND	3	KX130668	489,817	52,979	22.9	49.64	91	1	PV660462	SAMN48508521
LF82-10	3	ND	3	KX130668	437,638	52,895	341.8	49.62	91	1	PV660453	SAMN48508512
LF82-19	0	ND	3	KX130668	374,345	52,748	103.5	49.59	91	1	PV660457	SAMN48508516
LF82-23	0	*Bonnellvirus*	4	MN850624	469,574	42,378	33.1	55.76	54	0	PV660458	SAMN48508517

## Data Availability

The raw sequence reads were deposited to the NCBI SRA database under BioProject PRJNA1263023. BioSample and GenBank accessions are listed in Table 1 for each phage.
